# Anti-Inflammatory Effects of the Bioactive Compound Ferulic Acid Contained in *Oldenlandia diffusa* on Collagen-Induced Arthritis in Rats

**DOI:** 10.1155/2014/573801

**Published:** 2014-05-04

**Authors:** Hao Zhu, Qing-Hua Liang, Xin-Gui Xiong, Jiang Chen, Dan Wu, Yang Wang, Bo Yang, Yang Zhang, Yong Zhang, Xi Huang

**Affiliations:** ^1^Institute of Combined Traditional Chinese and Western Medicine, Xiangya Hospital, Central South University, Changsha, Hunan 410008, China; ^2^Center of Telemedicine, Xiangya Hospital, Central South University, Changsha, Hunan 410008, China

## Abstract

*Objectives*. This study aimed to identify the active compounds in *Oldenlandia diffusa* (OD) decoction and the compounds absorbed into plasma, and to determine whether the absorbed compounds derived from OD exerted any anti-inflammatory effects in rats with collagen induced arthritis (CIA). *Methods*. The UPLC-PDA (Ultra Performance Liquid Chromatography Photo-Diode Array) method was applied to identify the active compounds both in the decoction and rat plasma. The absorbable compound was administered to the CIA rats, and the effects were dynamically observed. X-ray films of the joints and HE stain of synovial tissues were analyzed. The levels of IL-1**β** and TNF-**α** in the rats from each group were measured by means of ELISA. The absorbed compound in the plasma of CIA rats was identified as ferulic acid (FA), following OD decoction administration. Two weeks after the administration of FA solution or OD decoction, the general conditions improved compared to the model group. The anti-inflammatory effect of FA was inferior to that of the OD decoction (*P* < 0.05), based on a comparison of IL-1**β** TNF-**α** levels. FA from the OD decoction was absorbed into the body of CIA rats, where it elicited anti-inflammatory responses in rats with CIA. *Conclusions*. These results suggest that FA is the bioactive compound in OD decoction, and FA exerts its effects through anti-inflammatory pathways.

## 1. Introduction

Rheumatoid arthritis (RA) is a heterogeneous systemic autoimmune disease with the main clinical manifestation of symmetrical panarthritis. Currently, there is a lack of effective cure or prevention [1]. Up to now, the cause and pathogenesis of this disease have been unclear. Accumulating evidence indicates that certain cytokine interleukins, such as tumor necrosis factor (TNF) alpha, could be induced and synergized to promote the induction of IL-1*β* and IL-6 in target cells [2], culminating in the production of factors such as matrix metalloproteinases and reactive oxygen species that drive erosive arthritis [3]. Consistent with the role of inflammatory cytokines in the pathogenesis of RA, the treatment of RA has largely focused on these cytokines, with the development of biological agents targeting inflammatory cytokines such as TNF [4]. The single-target method applied by Western medicine has faced many challenges. Chinese traditional medicine could be more effective due to its multitarget approach [5]. bizhongxiao decoction (BZXD), an empirical formula based on long-term RA treatment in our ward, has been shown to be very effective [5]. This formula was formed by combining several Chinese traditional herbs, including* Oldenlandia diffusa* [6]. It has been reported that the high levels of TNF-*α* and IL-1*β* typically found in patients with RA can be significantly reduced by the administration of BZXD [7, 8]. In addition, BZXD appears to have a positive effect on RA patients by inhibiting joint bone destruction and protecting joint function [6]. Because BZXD contains multiple herbs, in order to learn about this formula, we studied the herbs individually, starting with the most important one.* Oldenlandia diffusa* (OD) is the principal component of BZXD, and it has a very important medicinal role. Its pharmacodynamic properties have been the focus of research in traditional Chinese medicine (TCM) [9]. Previous studies have indicated that OD contains many chemicals, including triterpenoids, ferulic acid, sterols, iridoid glycosides, polypeptides, flavonoids, ursolic acids, oleanolic acids, and polysaccharides [10], some of which have multiple effects such as anti-inflammatory, antioxidative, and immunoregulatory [11, 12]. Sodium ferulate has been reported to have a beneficial effect on adjuvant arthritis treatment by reducing the level of IL-15 and IL-23 [13] and to have a curative effect on RA by influencing the expression of serum VEGF and TNF-*α* [14]. However, it is unknown which of the above compounds can be absorbed into the blood, resulting in a high false-positive rate. Therefore, to clarify the pharmacodynamic basis of OD's antirheumatism effect, we believe it is necessary to first identify the compounds absorbable in rat plasma by tracing the pathway of the compounds using a research strategy based in bioethnopharmaceutical analytical pharmacology [15].

## 2. Materials and Methods

### 2.1. Using the UPLC-PDA Method, We Identified the Active Compounds in OD and the Compounds Absorbable in Rat Plasma after Intragastric Administration of OD Decoction to Rats

The purity of all reference compounds was >99%. Acetonitrile and methanol (HPLC grade) were obtained from the Tedia Company, Inc, Fairfield, Ohio (USA). The Chinese herbal drug OD was purchased from the TCM Dispensary of Xiangya Hospital of Central South University. First, the drug was ground into a powder, then purified water was added to it in a ratio of 1 : 8. Next, the mixture was boiled twice for 30 min each; the mixture was filtered to extract the liquid; low-pressure rotary evaporation was carried out at a temperature of 60°C to get the concentrated OD decoction. The liquid was then processed into freeze-dried powder with a freeze dryer, with a yield rate of 18.56%, and the freeze-dried powder was sealed and stored in a refrigerator at a temperature of 4°C for future use. Ursolic acid, oleanolic acid, kaempferol, p-coumaric acid, FA, rutin, scopolamine lactone, and caffeic acid controls were purchased from Shanghai Yuanye Bio-Technology Co, Ltd, China. The purity of all reference compounds was >99%. The rats were with a body mass of 150 ± 30 g, provided by the Laboratory Animal Center of Hunan Provincial People's Hospital. The rats were fed ad lib and were exposed to a 12 h/12 h light/dark cycle (lighting time: 6:00–18:00). The background noise was 40 ± 10 dB, and the ambient temperature was 20 ± 3°C during a one-week adaptation period.

Chromatography column: ACQUITY UPLC BEH C18 (2.1∗50 mm 1.7 *μ*m); mobile phase: acetonitrile (A) and 0.5% acetic acid solution (B); detection wavelength: 190–400 nm; flow rate: 0.5 mL/min; column temperature: 40°C; sample volume: 5 *μ*mL; analysis time: 15 min. The OD test solution was injected into the device for detection. We also examined the linear relationship of the test solution and the day-to-day and intraday precision, stability, and repeatability, as well as the sample recovery rate.

Although OD contains many more chemical components, we have identified the following 8 compounds, through a literature search, as mainly reported: ursolic acid, oleanolic acid, kaempferol, p-coumaric acid, FA, rutin, scopolamine lactone, and caffeic acid. All of these substances were placed in methanol for ultrasonic dissolving to obtain the control stock solutions, the concentrations of which were 0.326 mg/mL, 0.334 mg/mL, 0.413 mg/mL, 0.294 mg/mL, 0.32 mg/mL, 0.336 mg/mL, 0.22 mg/mL, and 0.464 mg/mL, respectively. All of the solutions were sealed and then stored in a refrigerator at a temperature of 4°C. Purified water was used to ultrasonically dissolve the freeze-dried OD powder, which was then centrifuged and filtered to obtain the test solution of OD at a concentration of 86.22 mg (crude drug)/mL. The mother solution was then mixed with all of the 8 references at a ratio of 1 : 1 : 1 : 1 : 1 : 1 : 1 : 1.

Normal Sprague-Dawley (SD) rats were divided into an OD intragastric administration group and a control group. The rats in the OD group were administered the OD decoction intragastrically at a dose of 1.35 g/kg (crude drug/weight) (converted according to the body surface area of an adult human weighing 70 kg [16]); the rats in the control group were administered purified water intragastrically at the same volume. After 3 days of intragastric administration, the rats were fasting for 12 h. This was followed by a final administration, after which the rats were anesthetized and decapitated, and their plasma was extracted. A pipette was used to remove 2 mL of plasma and mixed evenly with 4 mL of acetonitrile, 2 mL of ethyl acetate, and 1.2 mL of acetone, in that order, to precipitate protein and extract the active compound. The mixture was then dissolved ultrasonically for 20 min and centrifuged at a speed of 3000 r/min for 20 min. Next, the supernatant was pipetted and blow-dried with nitrogen gas in a water bath at room temperature then redissolved with 50 *μ*L of acetic acid solution (20%) and 50 *μ*L of methanol and centrifuged at a speed of 12000 r/min for 20 min. The supernatant was pipetted once again, and the plasma sample was set aside to be analyzed. Exactly 50 *μ*L of plasma sample was injected into the UPLC system for analysis.

We also anesthetized and decapitated the rats from control group and extracted their plasma. The plasma were processed following the method above then divided in duplicate. For a better accuracy and reliability of the results, plasma A was tested as blank plasma. Plasma B was added with mother solution and then tested as positive control plasma.

### 2.2. Selection of Concentrations of FA for Intragastric Administration

Based on the clinical dose of OD and the FA content of OD, the concentration of FA administrated to rats was adjusted to match the equivalent human dose [16]. Another 4 concentrations were also used, which were half, two times, four times, and eight times the converted concentration. Therefore, a total of 5 intervention doses were administered, namely, 0.32, 0.64, 1.28, 2.56, and 5.12 *μ*g/g/d, which were, respectively, dose 1, dose 2, dose 3, and dose 4 groups. The purpose was to find the optimal dose of FA to intervene CIA.

25 of 30 healthy male SD rats were randomly selected for the purpose of CIA model replication, according to the modeling procedure of Chondrex. Bovine collagen type II (BIIC) solutions containing CFA or IFA were prepared and subcutaneously injected in the tail root, back, and sole of each rat on the 1st and 7th days, respectively. The other 5 rats were designed as control group.

Fourteen days after the immunization injection, all rats received intragastrically administered FA. The rats in the model group were intragastrically injected with purified water, while those in the normal group were permitted to drink freely.

The rats were anesthetized and decapitated on day 42, and the levels of IL-1*β* and TNF-*α* in their serum were measured to select the optimal dose of FA for intervention in the CIA rats. Statistical analysis of all data was accomplished with the SPSS 15.0 software package.

### 2.3. Effect of the Optimal Dose of FA on CIA Rats

Eighty SD rats (half male, half female) were randomly divided into 2 groups: normal (*n* = 20) and model (*n* = 60) groups. The 60 rats belonging to the latter group were assigned to CIA model replication. Two weeks after the immunization injection, animals failing in model replication were eliminated. Then, the remaining rats in model replication were subdivided into 3 groups, namely, the OD group, the FA group, and the model group.

The OD group received intragastrically administered OD decoction at a dose of 1.35 g/kg 2 times per day. The rats in the FA group received intragastrically administered reference FA at the optimal dose of 1.28 *μ*g/g/d 2 times per day. The rats in the model group received intragastrically administered purified water at the same volume. Those in the control group were fed normally.

### 2.4. Observation of General Conditions

During a period of 42 days, each rat was weighed every 2 weeks and was recorded. In 28th day, we compared with the record 14 days ago, which was in the 14th day; in the 42nd day, we compared with the record 14 days ago, which was in 28th day. After that, we calculated the difference value. In addition, the arthritis index of each rat was observed and recorded by means of arthritis index integration [17]. A score of 0–4 was assigned to each CIA rat based on the degree of joint redness and swelling, as well as joint enlargement and deformity. Zero point indicates no arthritis, 1 point indicates mild swelling after appearance of red spots, 2 points indicate moderate swelling of joints, 3 points indicate severe swelling of joints, and 4 points indicate severe swelling of joints and inability to bear weight. Every week before and after immunization, a pair of compasses (fine-angle) and a flexible ruler were used to measure the thickness (in mm) of the right rear foot of each rat [18]. Statistical analysis of all data was accomplished with SPSS 15.0 to evaluate whether there were significant differences among each group in 28th and 42nd days.

Radiographic evaluation of the joints of rats in each group was carried out every 2 weeks to observe the degree of joint destruction. With the help of the Radiology Department of Xiangya Hospital, we used a diagnostic (800 mA) X-ray apparatus (PHILIPS, Inc, USA) to take the radiographs. Under the condition of general anesthesia, we took frontal X-rays of both lower extremities of the rats.

On the 14th, 28th, and 42nd days after immunization, under general anesthesia with 10% chloral hydrate, we amputated the bilateral knee joint and the whole rear paw, including the ankle joint then fixed them in 10% neutral buffered formalin for 24 hours. They were decalcified in 14% EDTA decalcifying fluid for 5 days then neutralized in 5% sodium thiosulfate for 3 hours. They were then washed for 12 hours, embedded in dehydrated paraffin, and cut into 5-6 *μ*m slices (longitudinal). Next, the slices were placed into a 60°C oven for 30 min then soaked in xylene for 2 times 20 min. Next, they were soaked in 95% ethanol for 3 min, dipped into 80% ethanol for 1 min, and then in distilled water for 1 min. Then, they were dyed with hematoxylin for 15 min and washed, differentiated with acid alcohol for 3 s, washed with tap water for 10 min, dyed with eosin solution for 3 min, and washed again. Finally, we used 80% ethanol, 95% ethanol, and pure ethanol, successively, for gradient dehydration and mounted the samples with neutral gum. Pathological changes were observed under an optical microscope (Leica DFC425C) at 40x magnification.

The rats from the different groups were decapitated on the 28th or the 42nd days to sample their serum. Serum IL-1*β* and TNF-*α* levels were measured by enzyme-linked immunosorbent assay (ELISA; CUSABIO BIOTECH Co., Ltd, Wu Han, China) in pg/mL. The serum samples were centrifuged at 1000 r/min for 15 min and the serum was stored at −80°C. Two ELISA kits were utilized using a flat bottom with 96-well plates. Each well was coated with protein, and the serum was incubated in a well. After 1 hour at room temperature, the serum was removed and the samples were washed off with a series of buffer rinses. Next, enzymes (peroxidase) were added to metabolize the colorless substrates into colored products for 30 min, and the colored products were placed in the wells. When the enzyme reaction was complete, the entire plate was placed into a plate reader, and the optical density was determined for each well at 405 nm. The statistical analysis of all data was accomplished with SPSS 15.0 to evaluate whether there was a significant difference between day 28 and day 42.

## 3. Results

### 3.1. Identification of the Active Compounds in OD by UPLC-PDA

**Figure 1:**
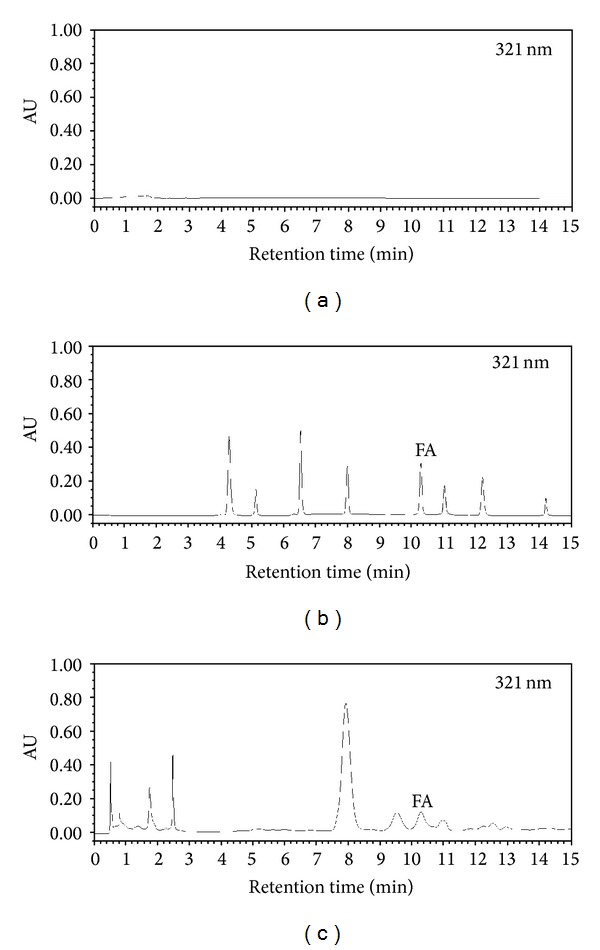
Typical chromatogram of samples in each group: (a) blank methanol; (b) eight reference compounds; (c) test solution of OD freeze-dried powder; FA refers to ferulic acid. All of the samples were scanned under the wavelength of 321 nm.

Exactly 50 *μ*L of mother solution and test solution (86.22 mg crude drug/mL) was pipetted and injected into the UPLC system according to the chromatography procedure described above. The mother solution was scanned by the PDA detector (wavelength: 190–400 nm) under 321 nm, and the results in Figure 1(b) showed that the 8 references were well separated. They were ursolic acid (223 nm), oleanolic acid (224 nm), kaempferol (366 nm), p-coumaric acid (308 nm), FA (321 nm), rutin (255 nm, scopolamine lactone (228 nm), and caffeic acid (325 nm). FA was well separated at the wavelength of 321 nm after 10.29 min. On the OD test samples as shown in Figure 1(c), maximum absorption of FA was detected at the wavelength of 321 nm after 10.26 min. On the basis of these results, compared with blank methanol in Figure 1(a), we may conclude that FA is present in OD, and it is one of the chemical compounds in OD.

### 3.2. Detection of Absorbable Compounds in the Plasma of Rats Receiving Intragastrically Administrated OD Decoction by UPLC-PDA

**Figure 2:**
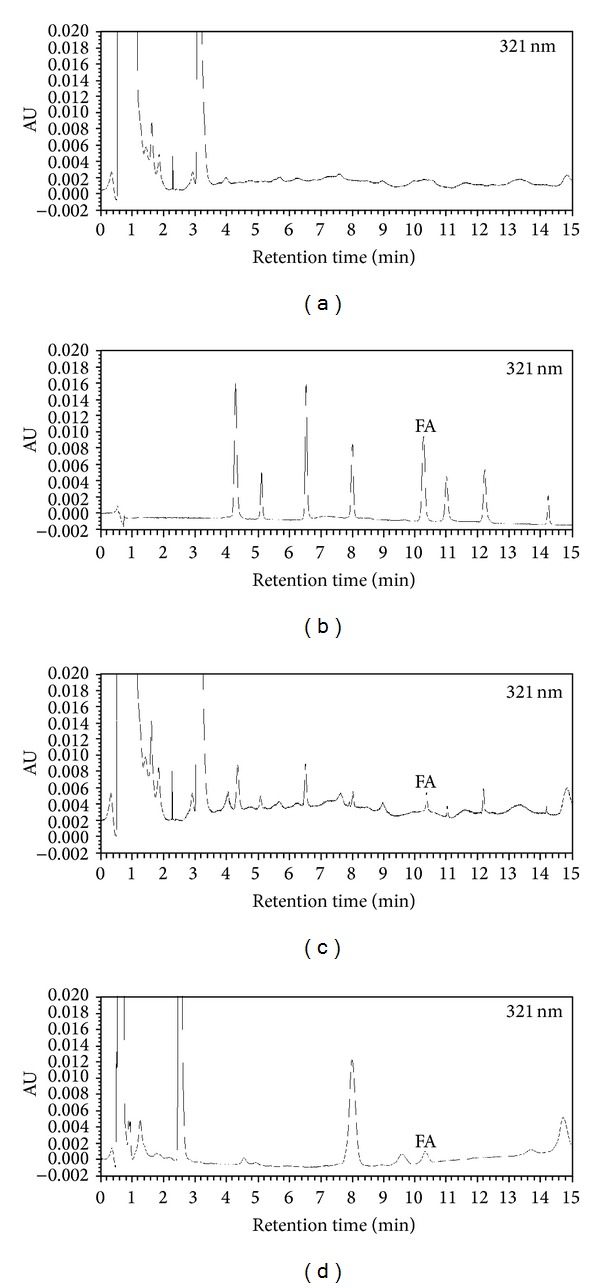
Typical chromatogram of samples in each group: (a) blank plasma; (b) eight reference compounds; (c) positive control; (d) plasma from rat following intragastrically administrated with OD freeze-dried powder; FA refers to ferulic acid. All of the samples were scanned under the wavelength of 321 nm.

Chromatography conditions were the same as tested above. As show in Figure 2(b), FA in mother solution achieved good separation at 321 nm after a time of 10.25 min. After orally administering OD to the rats, the plasma was tested, as show in Figure 2(d); maximum absorption of FA occurred at the wavelength of 321 nm after 10.29 min. Compared with blank plasma in Figure 2(a) and positive control in Figure 2(c), we found that FA could be detected in plasma of rats administrated with OD, not in blank plasma. Thus, we may safely conclude that FA could be absorbed into the blood circulation of rats, as a bioactive compound in OD. We believe that the 2 critical elements contributing to the results were the concentration of plasma and the method of preparation, which were based on a number of preliminary experiments.

### 3.3. Selection of the Optimal Dose of FA for the CIA Rats

**Figure 3:**
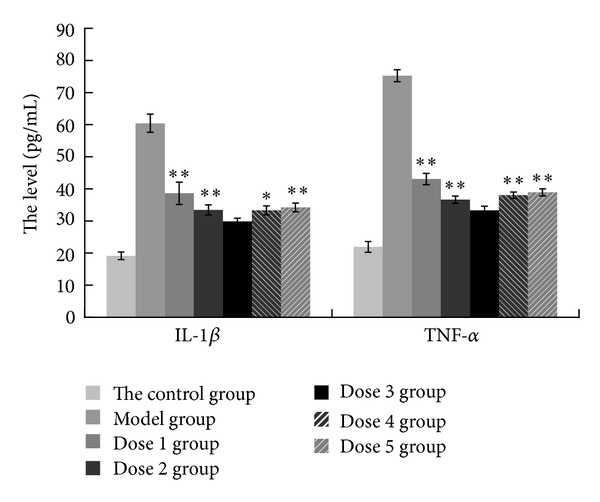
ELISA analysis of IL-1*β* and TNF-*α* in plasma of CIA rats following 5 different doses of FA (ferulic acid). And the control group and model group were also tested. The values in each group are represented as means ± SD. Statistics showing for significant difference compared with dose 3 group were marked (**P < 0.01).

Figure 3 shows the levels of IL-1*β* and TNF-*α* in rat serum in each group: on the 42nd day, the levels of IL-1*β* and TNF-*α* in the model group were 60.41, 75.22 pg/mL, and both increased compared with the normal group, which were 19.15, 21.93 pg/mL; the levels of IL-1*β* and TNF-*α* after intervention both decreased in each group compared with the model group (*P* < 0.01); among all of the intervention groups, the levels of IL-1*β* and TNF-*α* were 29.81, 33.26 pg/mL and appeared to be the lowest in dose 3 group. Compared to dose 3 group, the levels of IL-1*β* and TNF-*α* in other dose groups were significantly higher (*P* < 0.01). According to our results, dose 3 group had the greatest effect in decreasing the level of IL-1*β* and TNF-*α*. Thus, we can conclude that the dose in 1.28 *μ*g/g/d was the optimal dose of FA for the intervention.

### 3.4. Effect of Optimal FA and OD Decoction Dose on CIA Rats

**Figure 4:**
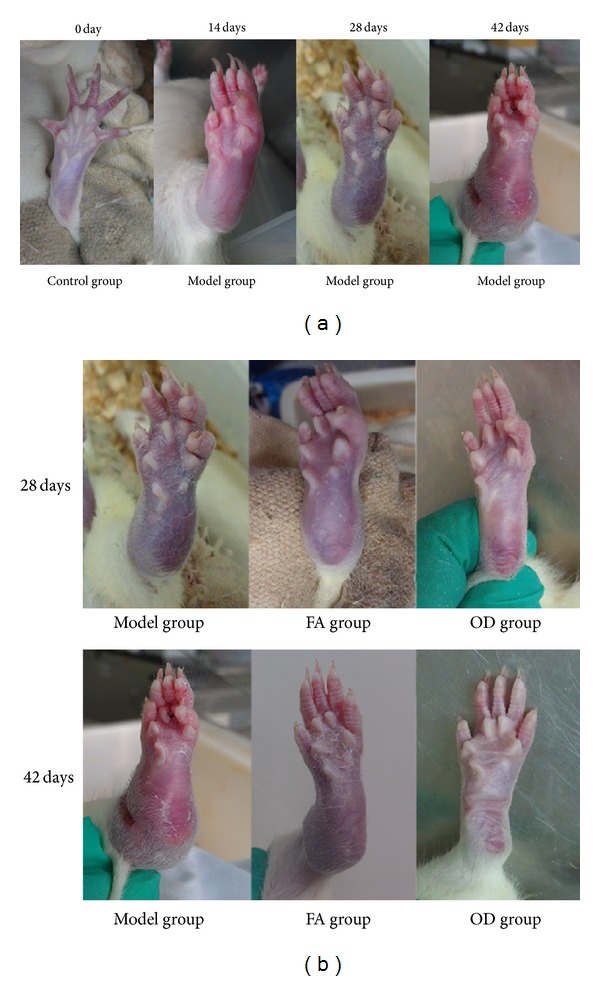
General conditions of joints from 3 groups from day 0 to day 42, including the model group, FA group, OD (*Oldenlandia diffusa*) group. (a) represents the model group every 14 days. (b) represents 3 groups every 14 days.

The signs of arthritis appeared in the experimental rats 5–7 days after immunization. As shown in Figure 4, on the 14th day, the ankle and toe joints of rats in the model group showed more obvious redness, swelling, and hyperemia than before; redness, swelling, and hyperemia were simultaneously observed at both upper limbs of some rats. The rats in the model group were evaluated at that time; the replication rate of the CIA model reached 95%. On the 28th day, the rats in the model group were inactive and drowsy, ate and drank less, and reacted slowly when the cage was disturbed. The swelling of joints was more severe than before, and some rats had subcutaneous ecchymoses. Regarding the rats in the FA and OD groups, redness and swelling of joints of both lower limbs were reduced; hyperemic and swollen skin at the heels showed slight shrinkage; red spots and ecchymoses of joints were reduced compared to the model group. However, more pronounced changes of this kind were observed in the OD group. On the 42nd day, rats in the model group showed matted hair and increased joint symptoms; both lower limbs of some rats had reduced movement or complete loss of movement. In the FA group, redness and swelling of the joints were gradually alleviated, red spots and ecchymoses of joints faded away, and hyperemic and swollen skin at the heels showed obvious shrinkage. In the OD group, the decrease in redness and swelling of joints was more obvious than in the FA group; red spots and ecchymoses of joints also faded away, and the shrinkage on the shin was much greater than in the FA group (see Figure 4 for all the changes). On the 42nd day, compared with model group, there were no obvious redness, swelling, or hyperemia of the paw in the OD group, nor red spots or ecchymoses, only some slight skin shrinkage could be observed. All the pictures recorded in different times were shown in Figure 4.

**Figure 5:**
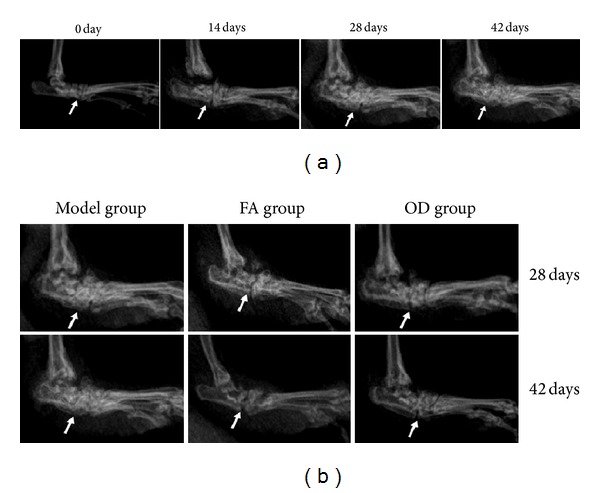
X-ray films of joints from 3 groups every 14 days. (a) represents the model group from day 0 to day 42. (b) represents 3 groups every 14 days. The joint change in each group was marked with white arrow.

The X-ray images showed some changes. In Figure 5(a), the control group had no swelling of soft tissues around the ankle joints, and the toe joint spaces were clear. On the 14th day, the X-ray films of rats in the model group showed swelling of tissues surrounding the ankle joints, but the joint spaces were still clear. On the 28th day, vague and narrow toe joint spaces of some rats in model group were observed, when it came to the 42nd day, the joint spaces was still unclear and even aggravated. See the change pointed out by the white arrow. In Figure 5(b), there were obvious changes between model group and FA, OD groups. When it came to the 42nd day, see the places pointed out by the white arrow in Figure 5(b), we observed that the swelling of soft tissues in OD group reduced, in comparison with the model group. And the ankle joint spaces in the FA and OD group were slightly more visible than in the model group, especially the OD group, but not as clear as in the control group. These results may indicate that both of FA and OD have the curative effect on swelling and arteriostenosis in CIA, and the OD' effect was more obvious than FA.

**Figure 6:**
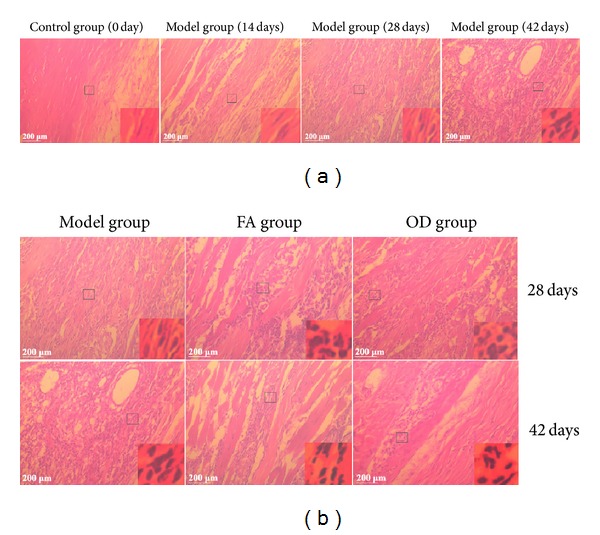
HE staining of joints from 3 groups every 14 days. (a) represents the model group from day 0 to day 42. (b) represents 3 groups every 14 days. At the left below of all HE pictures, a scale bar of 200 *μ*m was marked; at the right below, a magnified view was shown 14 times enlarged from the original.

In HE staining test, as shown in Figure 6, the black spots refer to inflammatory cell infiltration. In Figure 6(a), no obvious abnormality was found in synovial tissues in the control group. Fourteen days after immunization, some inflammatory cell infiltration was observed in the synovial tissues of rats in the model group. On the 28th and 42nd days, a large number of inflammatory cells could be seen in the model group. In Figure 6(b), on the 28th day, no obvious inflammatory cell infiltration difference was observed among model and FA and OD groups. On the 42nd day, inflammatory cell infiltration in FA and OD groups was alleviated compared with 28th day. At the lower left of all HE pictures, a scale bar of 200 *μ*m was marked; at the lower right, a magnified view was shown 14 times enlarged from the original. These results may indicate that FA and OD have the effect of alleviating inflammatory cell infiltration.

**Figure 7:**
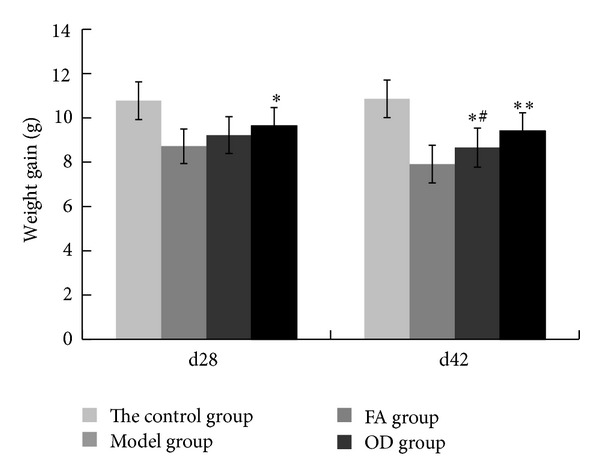
Weight gain records of 4 groups on 28th day and 42nd day, including control, model, FA, and OD groups. The values in each group are means ± SD. In 28th day and 42nd day, statistics in OD group are shown for significant difference (*P < 0.05, **P < 0.01) compared with the model group. In 42nd day, statistics in FA group are shown for significant difference (^#^ *P* < 0.05) compared with the OD group.

Each rat was weighted every 2 weeks. In the 28th day, we compared with the record in 14th day; in 42nd day, we compared with the record in 28th day. And we recorded the difference value as weigh gain. As shown in Figure 7, in 28th day, the model group got the lowest value of 8.72 g. FA group got the value of 9.22 g, which had no significant difference compared with model group (*P* > 0.1). OD group got the value of 9.64 and had significant difference compared with model group (*P* < 0.01). In 42nd day, the model group got the lowest value of 7.91 g. FA group got the value of 8.66 g, which had significant difference compared with model group (*P* < 0.05). OD group got the value of 9.46 and had significant difference compared with model group (*P* < 0.01). In 42nd day, we compared FA group with OD group and found significant difference (*P* < 0.01). According to these results, rats in FA and OD group grew faster than model group, but it was obviously on 42nd day.

**Figure 8:**
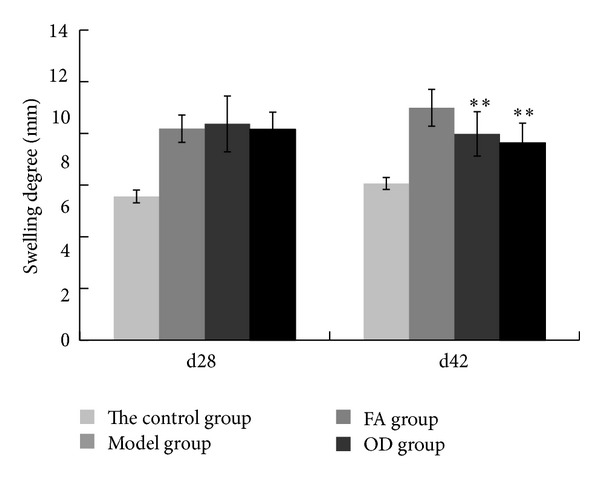
Degree of swelling of 4 groups on 28th day and 42nd day, including control, model, FA, and OD groups. The paws in each group are means ± SD. On 42nd day, statistics in the FA and OD groups are shown for significant difference (*P < 0.05, **P < 0.01) compared with the model group.

Assessment of the degree of paw swelling of rats in each group was carried out on 28th day and 42nd day. As shown in Figure 8, on 28th day, FA group got the swelling degree of 8.1 mm, and OD group was 7.45 mm. Compared with the model group of 8.48 mm, there was no significant difference (*P* > 0.2). On 42nd day, the degree of paw swelling in the FA and OD groups showed a downtrend compared with the model group, and the difference was statistically significant (*P* < 0.01); FA got 7.76 mm, OD got 6.92 mm, and the model group got 8.99 mm. Meanwhile, neither on 28th day nor 42nd day was there significant difference compared between FA group and OD group (*P* > 0.08). These results may indicate that both FA and OD had the effect of detumescence in CIA.

**Figure 9:**
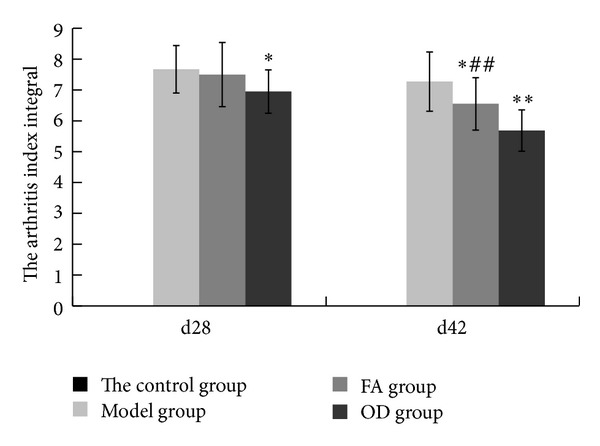
Arthritis index records of 4 groups on 28th day and 42nd day, including control, model, FA and OD groups. The values in each group are means ± SD. Statistics in the FA and OD groups are shown for significant difference (*P < 0.05, **P < 0.01) compared with the model group. On 42nd day, statistics in the FA group are shown for significant difference (^##^ *P* < 0.01) compared with the OD group.

As shown in Figure 9, on both 28th day and 42nd day, the control group got zero degree of arthritis index. As the immune time progressed, the arthritis index integrals of the 3 groups showed an uptrend. On 28th day, the index in the model group was 8.06. FA group got 7.5. OD group got 6.95. Compared with model group, OD group had significant difference (*P* < 0.01). On 42nd day, the downtrend in FA and OD groups was significantly greater compared with the model group (*P* < 0.01); FA group got the degree of 6.55, OD group got 5.68, and model group got 7.27, and the decrease in the FA group was much more pronounced than in the OD group (*P* < 0.01).

**Figure 10:**
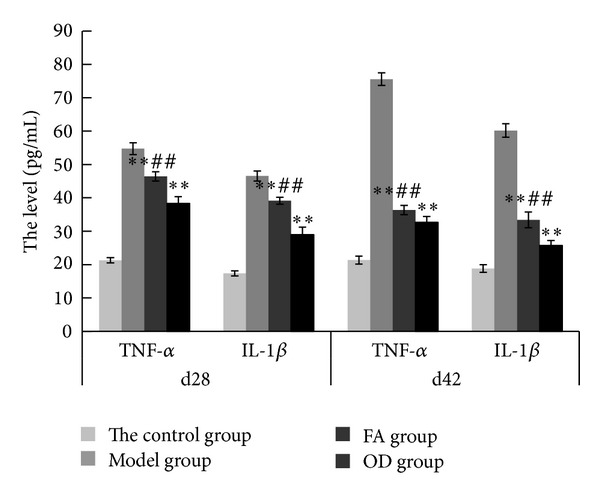
ELISA records of 4 groups on 28th day and 42nd day, including control, model, FA, and OD groups. The values in each group are represented as means ± SD. Statistics in the FA and OD groups are shown for significant difference (**P < 0.01) compared with the model group. Statistics in the FA group are shown for significant difference (^##^ *P* < 0.01) compared with the OD group.

The ELISA test of the serum of rats in each group was performed on the 28th and 42nd day to detect the inflammatory cytokines in serum (see Figure 10 for the levels of IL-1*β* and TNF-*α* in the rat serum in each group). On 28th day, the levels of IL-1*β* and TNF-*α* in the serum of the FA group were 39.13 pg/mL and 46.44 pg/mL, compared with the model group of 46.56 pg/mL and 54.74 pg/mL; FA group showed a significant downtrend (*P* < 0.01); the levels of IL-1*β* and TNF-*α* in the serum of the OD group were 32.69 pg/mL and 38.31 pg/mL, compared with the model group; OD group showed a significant downtrend (*P* < 0.01). On 42nd day, the levels of IL-1*β* and TNF-*α* in the serum of the FA group were 33.40 pg/mL and 36.33 pg/mL, compared with the model group of 60.19 pg/mL and 75.58 pg/mL, FA group showed a significant downtrend (*P* < 0.01); the levels of IL-1*β* and TNF-*α* in the serum of the OD group were 25.76 pg/mL and 28.97 pg/mL, compared with the model group; OD group showed a significant downtrend (*P* < 0.01). Interestingly, on both 28th day and 42nd day, the level of inflammatory cytokines in the FA group was higher than in the OD group, and the difference was statistically significant (*P* < 0.05). This result may indicate that both FA and OD had the effect of reducing the level of IL-1*β* and TNF-*α* in CIA, and OD was more effective than FA.

## 4. Discussion

In clinical therapies, traditional Chinese medicines are mostly orally administered, and the bioactive compounds contained in them can take effect only after being absorbed into the blood [19]. Accordingly, it was necessary to explore the absorbable bioactive compounds. Previously, HPLC has been frequently applied in the quality control of OD [20], and the compounds detected were mostly triterpenoids, sterols, iridoid glycosides, polypeptides, flavonoids, ursolic acids, oleanolic acids, and polysaccharides [12]. However, the metabolic processes involved in the biological action of OD have not been studied. In contrast to previous studies, which used the HPLC-method, we used the UPLC-method. UPLC significantly reduces the analysis time and solvent consumption and improves the flexibility and separation efficiency [21]. As a result, we have been able to identify the absorbable bioactive compounds contained in OD and found that FA could be absorbed into the blood of rats. As one of the mainstream approaches to study traditional Chinese medicine, this method paved a road for the following research.

In the pathological process of CIA, inflammatory cells produce reactive oxygen metabolites such as superoxide anions (e.g., O^2−^), which induce lipid peroxidation and biomembrane damage [22] and produce inflammatory cytokines such as TNF-*α* and IL-1 at the same time. These substances destroy articular cartilage [23] and mediate a series of pathological reactions such as arthrocele and joint deformity [24]. As shown in Figures 4 and 5, the condition of the model group deteriorated with time, and inflammatory cells and inflammatory cytokines increased as time progressed, as shown in Figures 6 and 10. Thus, the levels of TNF-*α* and IL-1*β* in plasma may reflect the activity of inflammatory cells [25]. A previous report has indicated that FA had the ability to decrease the levels of hydrogen peroxide-induced IL-1*β*, TNF-*α*, MMP-1, and MMP-13, thereby reducing bone destruction [26], synovitis, and the erosion of cartilage in antigen-induced arthritis [27]. Because of their purported protective effects on joints, FA and OD (which contains FA) were administered to CIA rats, and, as shown in Figures 4–10, the animals' symptoms ware ameliorated, the number of inflammatory cells decreased, and the levels of IL-1*β* and TNF-*α* declined by day 28 and day 42. Both FA and OD demonstrated an anti-inflammatory effect on collagen-induced arthritis. It should be noted that a comparison of the treatment effects of FA and OD indicated that the OD group had a better therapeutic outcome, especially regarding general conditions and the level of inflammatory cytokines.

Figure 4(b) shows that the paws of the rats in the OD group had less swelling and hyperemia than those in the FA group, on both day 28 and day 42. Furthermore, statistical analysis revealed that the arthritis index was lower in the OD group on both day 28 and day 42, as shown in Figure 9. Moreover, Figure 10 shows that the levels of IL-1*β* and TNF-*α* in the OD group were lower than in the FA group on both day 28 and day 42 (*P* < 0.01). These results show that the OD group experienced a better curative effect than the FA group. We suggest that the reason for this is that OD contains multiple bioactive compounds, which act through multitarget pathways. Although our study has only demonstrated that FA was one of the bioactive compounds in OD, previous research has indicated that ursolic acid extracted from OD has a suppressive effect on rheumatoid arthritis by inhibiting paw swelling and plasma PGE(2) production [28]. In addition, hentriacontane, one of the constituent compounds of OD, exerts its anti-inflammatory effect through the regulation of the activation of nuclear factor-*κ*B and caspase-1 [29]. Furthermore, both ursolic acid and oleanolic acid have demonstrated free radical scavenging activity [30]. Thus, OD's protective effect on joints appears to occur through multiple ways due to the various antiarthritis effects exerted by its constituent bioactive compounds. Since FA is just one of several bioactive compounds contained in OD, it exerts its effect through fewer pathways relative to OD. This can explain why OD has a better therapeutic effect than FA.

## 5. Conclusion

All of the above results may be related to the anti-inflammatory effects of FA contained in OD. Since FA is just one of several bioeffective compounds contained in OD, our results indicate that OD could have a better anti-inflammatory effect on the symptoms of CIA than FA. As shown in Figures 4–10, all of the results from the OD group were better than FA group, indicating that there may be several pathways through which OD exerts its effects. Such multitarget pathways may be the reason why traditional Chinese medicine has shown to be more effective than single-target Western medications in the treatment of CIA. The findings of this study offer a new direction for further studies of the pharmacodynamic bioactive compounds in OD and BZXD.

## Abbreviations

OD: * Oldenlandia diffusa*
FA: Ferulic acid
CIA: Collagen induced arthritis
UPLC-PDA: Ultra performance liquid chromatography photo-diode array
RA: Rheumatoid arthritis
BZXD: Bizhongxiao decoction.
